# Thrombotic Occlusion in Coronary Ectasia Presenting as STEMI

**DOI:** 10.1016/j.jaccas.2025.105769

**Published:** 2025-10-22

**Authors:** Mohammed Ali, Ramkumar Narendran, Faisal Hasan

**Affiliations:** a Rush Medical College, Chicago, Illinois, USA; b Rush University Medical Center, Chicago, Illinois, USA

**Keywords:** coronary angiography, coronary vessel anomaly, myocardial infarction

## Abstract

**Background:**

Coronary artery ectasia (CAE) is a rare vascular abnormality characterized by diffuse coronary dilation. It poses a risk for thrombus formation and acute coronary events owing to disrupted flow dynamics.

**Case Summary:**

A 61-year-old man with aortoiliac aneurysms, dyslipidemia, and hypertension presented to the emergency room with chest pain and elevated troponins. Coronary angiography revealed diffuse ectasia with heavy thrombus burden in the right coronary artery. Partial thrombectomy and glycoprotein IIb/IIIa inhibitor therapy were administered. Follow-up angiography showed an organizing thrombus. The patient was treated with triple antithrombotic therapy and was discharged in stable condition.

**Discussion:**

This case highlights the thrombotic complications of CAE and reviews strategies for diagnosis, risk stratification, and therapeutic interventions.

**Take-Home Message:**

Coronary artery ectasia can present as acute coronary syndrome due to thrombus formation. Treatment often requires both catheter-based intervention and aggressive medical therapy.

## Graphical Abstract

**Figure:**
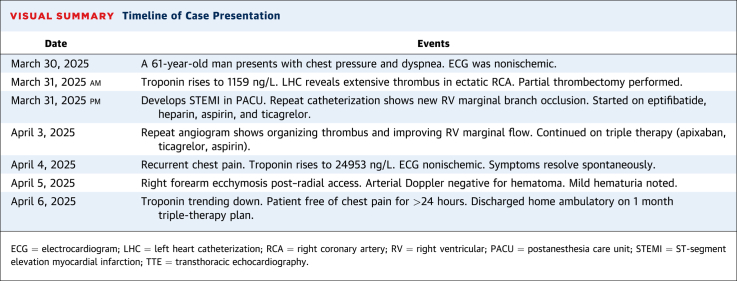

## History of Presentation

**Figure 1:**
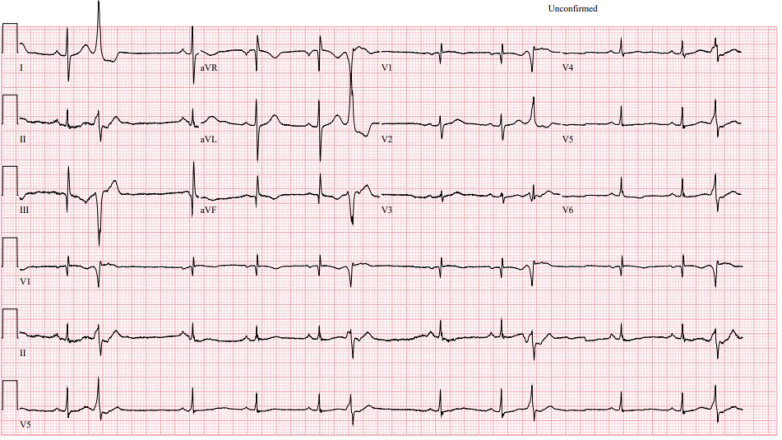
Initial Electrocardiogram With Premature Ventricular Complexes With Evidence of Previous Inferior Infarct

A 61-year-old man with history of hypertension, dyslipidemia, abdominal aortic aneurysm, and bilateral iliac artery aneurysms presented with chest pressure and dyspnea. His symptoms were nonexertional and were not relieved by rest. Initial electrocardiogram showed no ischemic changes (Figure 1). High-sensitivity troponin levels trended upward, peaking on hospitalization day 2.

## Past Medical History

The patient's medical history included hypertension, dyslipidemia, gastroesophageal reflux disease, nephrolithiasis, gout, elevated prostate-specific antigen, and known abdominal aortoiliac aneurysms.

## Differential Diagnosis

Considerations included acute coronary syndrome (ACS), aortic dissection, pulmonary embolism, pericarditis, and noncardiac chest pain.

## Investigations

Electrocardiogram on arrival was nonischemic. Cardiac biomarkers were elevated. Coronary angiography revealed diffuse ectasia with extensive thrombus in the right coronary artery (RCA) and stenosis in the left anterior descending artery (2, 3, and 4).

**Figure 2:**
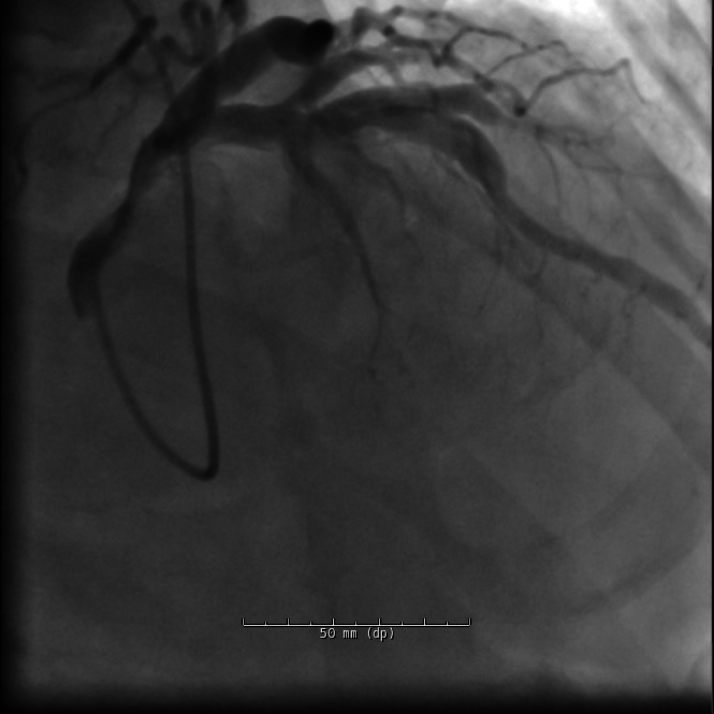
Cranial RAO View Revealing Moderate Mid-LAD Stenosis LAD = left anterior descending artery; RAO = right anterior oblique.

**Figure 3:**
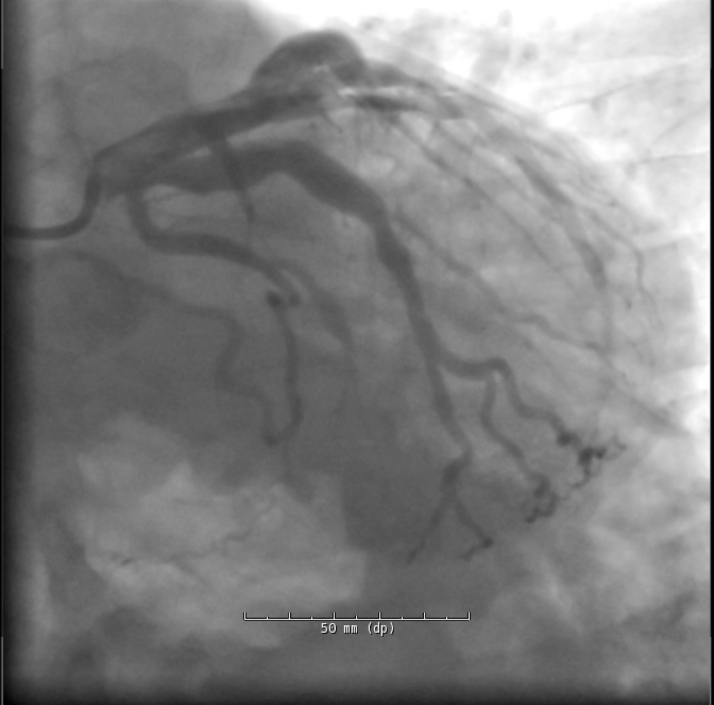
Hospitalization Day 2: LAO Caudal View Demonstrating Ectatic Left Coronary Artery With Bifurcation Into the LAD and LCx, and Distal LAD Stenosis LAD = left anterior descending artery; LAO = left anterior oblique; LCx = left circumflex artery.

**Figure 4:**
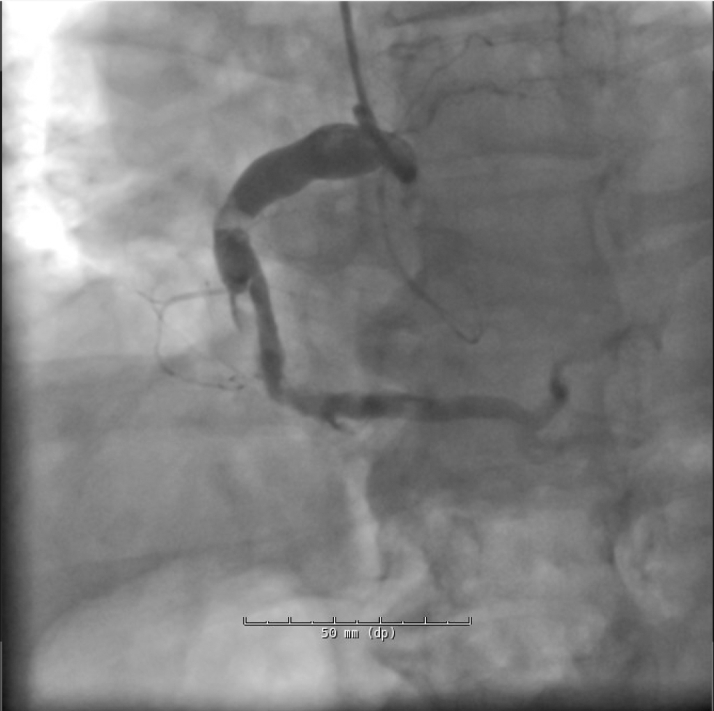
Hospitalization Day 2: RAO View of the RCA Showing Heavy Thrombus Burden and Proximal Stenosis RCA = right coronary artery; RAO = right anterior oblique.

## Management

On hospitalization day 2, the patient underwent left heart catheterization. Partial aspiration thrombectomy of the RCA was performed using the Penumbra CAT Rx system. Hours later, the patient experienced chest pain and ST-segment elevations in the anterolateral leads. Repeat catheterization revealed new right ventricular marginal branch occlusion. The patient received eptifibatide (Integrilin) infusion and was maintained on heparin, aspirin, and ticagrelor.

## Outcome and Follow-Up

**Figure 5:**
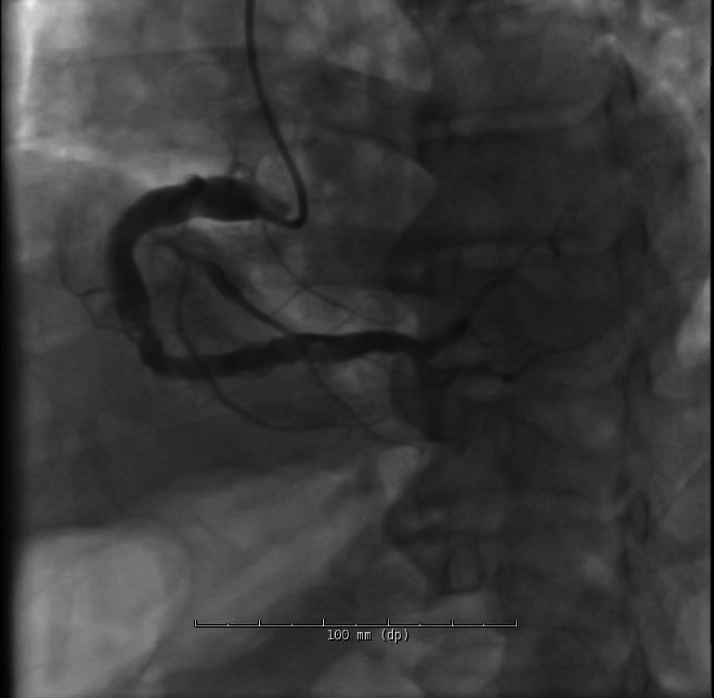
Hospitalization Day 6: RAO View of the RCA Showing Improved Blood Flow With No Blockage RCA = right coronary artery; RAO = right anterior oblique.

Repeat angiography on hospitalization day 6 showed organizing thrombus and improved right ventricular branch flow (Figure 5). The patient was continued on triple therapy including apixaban. On hospitalization day 7, troponin peaked again at 24953 ng/L during a transient chest pain episode. No new ischemic changes were seen. On hospitalization day 8, the patient developed forearm ecchymosis after radial access and required a Doppler to rule out forearm hematoma. On hospitalization day 9, troponin decreased to 9075 ng/L. The patient was discharged home, free of chest pain. Outpatient follow-up is planned with continued monitoring of symptoms and a 1-month course of triple therapy.

## Discussion

Coronary artery ectasia (CAE), defined as a dilation of the coronary artery segment exceeding 1.5 times the adjacent normal vessel diameter, represents a unique and often under-recognized subset of coronary artery disease.^1^ The condition has a reported prevalence ranging from 0.3% to 5% of angiographic studies and most commonly affects the RCA.^2^ Although it may coexist with obstructive coronary artery disease, CAE can also present in isolation, particularly in association with connective tissue disorders, vasculitides, or aneurysmal diathesis, as suspected in our patient with known aortoiliac aneurysms.^3^

The pathogenesis of CAE is multifactorial. In atherosclerotic CAE, excessive arterial remodeling is believed to be driven by chronic inflammation and enzymatic degradation of the extracellular matrix, primarily via matrix metalloproteinases (MMPs), especially MMP-3 and MMP-9.^4^ These enzymes contribute to medial thinning and elastic fiber breakdown, ultimately weakening the arterial wall. Systemic inflammatory conditions such as Kawasaki disease or Takayasu arteritis may also lead to similar structural changes. Given this mechanistic overlap, CAE may be seen as a coronary manifestation of systemic vascular remodeling.

From a hemodynamic standpoint, ectatic segments promote turbulent flow, delayed contrast clearance on angiography, and intraluminal stasis, all of which create a prothrombotic environment.^5^ This disrupted laminar flow leads to platelet activation and potential thrombus propagation within the dilated arterial segment. Intravascular imaging modalities such as intravascular ultrasound and optical coherence tomography can be valuable in identifying mural thrombus and eccentric plaques and in distinguishing true ectasia from pseudoaneurysms, although these tools were not used in our case given the acute setting.^4^

Clinically, CAE may present across a spectrum ranging from asymptomatic to life-threatening ACS. The ACS presentation in CAE poses specific challenges. Our case highlights how a thrombotic occlusion within an ectatic RCA, absent critical stenosis, can still precipitate a STEMI, consistent with the idea that flow-limiting thrombus—not just fixed narrowing—drives ischemia in these patients.

Management remains controversial, with no consensus guidelines given the rarity of CAE and lack of randomized clinical trials. Acute presentations are generally managed using principles extrapolated from conventional ACS care. Antiplatelet therapy with aspirin and P2Y12 inhibitors is standard, and the use of glycoprotein IIb/IIIa inhibitors—as in this case—has shown potential benefit in promoting thrombus resolution. The role of anticoagulation remains debated. Although chronic anticoagulation may reduce future thrombotic events, especially in large or sluggish-flow ectatic vessels, the bleeding risk is non-negligible.^6^ In our patient, the decision to initiate triple therapy (aspirin, ticagrelor, and apixaban) was guided by the high thrombotic burden and recurrent ischemic episodes despite dual antiplatelet therapy.

Percutaneous intervention in CAE is technically challenging. Balloon sizing and stent deployment can be complicated by vessel size heterogeneity and inadequate stent wall apposition, which increases the risk of stent thrombosis and embolization.^7^ Thus, percutaneous coronary intervention should be reserved for focal stenotic lesions or refractory thrombus where conservative therapy fails. Our case demonstrates the effectiveness of mechanical thrombectomy with the Penumbra CAT Rx system and pharmacologic support without requiring stent placement.

Surgical approaches, such as ligation of ectatic segments and bypass grafting, have been described but are reserved for patients with refractory angina or high-risk anatomy.^8^ These procedures carry significant morbidity and are generally considered a last resort.

Finally, the prognosis of CAE is variable and depends largely on coexistent coronary artery disease, the degree of vessel dilation, and the presence of thrombotic complications. Long-term follow-up should include aggressive risk factor modification, serial imaging when indicated, and patient education regarding symptoms of ischemia.

This case is noteworthy not only given the rarity of isolated CAE presenting with RCA thrombosis but also for the successful management using a combination of mechanical and pharmacologic therapies. It illustrates the importance of early recognition, individualized treatment planning, and the need for further clinical research to guide therapy in this under-represented population.

## Conclusions

This case illustrates how CAE can result in life-threatening thrombotic occlusion. Prompt recognition and intervention are essential. Treatment requires a multidisciplinary approach, balancing revascularization techniques with systemic anticoagulation. The absence of evidence-based guidelines highlights the need for further studies on management strategies tailored to CAE.

Surgical intervention, including bypass grafting or ligation of ectatic segments, is rarely performed and typically reserved for patients with persistent symptoms or recurrent thrombotic events despite medical therapy. Comparative studies are limited, but case reports show variable outcomes depending on vessel size, thrombus burden, and response to medical therapy. This case aligns with reports showing that short-term triple therapy can be safe and effective, especially when combined with careful procedural strategy.

## Funding Support and Author Disclosures

The authors have reported that they have no relationships relevant to the contents of this paper to disclose.

**Take-Home Messages**

• Coronary artery ectasia can present as acute coronary syndrome due to thrombus formation.
• Treatment often requires both catheter-based intervention and aggressive medical therapy.

**Visual Summary:** Timeline of Case Presentation

Date	Events
March 30, 2025	A 61-year-old man presents with chest pressure and dyspnea. ECG was nonischemic.
March 31, 2025 am	Troponin rises to 1159 ng/L. LHC reveals extensive thrombus in ectatic RCA. Partial thrombectomy performed.
March 31, 2025 pm	Develops STEMI in PACU. Repeat catheterization shows new RV marginal branch occlusion. Started on eptifibatide, heparin, aspirin, and ticagrelor.
April 3, 2025	Repeat angiogram shows organizing thrombus and improving RV marginal flow. Continued on triple therapy (apixaban, ticagrelor, aspirin).
April 4, 2025	Recurrent chest pain. Troponin rises to 24953 ng/L. ECG nonischemic. Symptoms resolve spontaneously.
April 5, 2025	Right forearm ecchymosis post–radial access. Arterial Doppler negative for hematoma. Mild hematuria noted.
April 6, 2025	Troponin trending down. Patient free of chest pain for >24 hours. Discharged home ambulatory on 1 month triple-therapy plan.

ECG = electrocardiogram; LHC = left heart catheterization; RCA = right coronary artery; RV = right ventricular; PACU = postanesthesia care unit; STEMI = ST-segment elevation myocardial infarction; TTE = transthoracic echocardiography.
